# Prevalence of depression in childhood-onset systemic lupus erythematosus: a systematic review and meta-analysis

**DOI:** 10.3389/fped.2026.1966337

**Published:** 2026-10-29

**Authors:** Xiao-Jing Xia, Min Pan

**Affiliations:** 1 Department of Pediatric Nephrology and Rheumatism and Immunology, Shandong Provincial Hospital Affiliated to Shandong First Medical University, Jinan, China; 2 Department of Pediatric Respiratory, Shandong Provincial Hospital Affiliated to Shandong First Medical University, Jinan, China

**Keywords:** childhood-onset systemic lupus erythematosus, depression, meta-analysis, prevalence, systematic review

## Abstract

**Objective:**

Childhood-onset systemic lupus erythematosus (cSLE) is frequently associated with psychological symptoms, particularly depression. However, there remains a lack of quantitative evidence regarding the prevalence of screening-positive depressive symptoms based on the currently available evidence. In this context, we conducted a systematic review and meta-analysis addressing this important issue.

**Methods:**

Three medical databases, including PubMed, Embase, Cochrane Library were utilized in this study. We included studies that reported on cSLE patients experiencing depression for a meta-analysis, without publication language restrictions. Anxiety were included as secondary outcomes. STATA software were employed to synthesize the results as well as to assess heterogeneity and publication bias.

**Results:**

Totally 12 eligible studies encompassing 652 patients were incorporated into this meta-analysis. Each of the publications included exhibited commendable methodological rigor, with Newcastle-Ottawa scores no less than 6. The pooled prevalence was [32.6%] (95% CI: [24.5%–41.8%]). Between-study heterogeneity was moderate (I^2^ = [73.66%]; *τ*^2^ = [0.26] on the logit scale). Subsequent subgroup analysis indicated that the prevalence of depression varied according to the different screening tools used: it was found to be 26% with CDI (95% CI: 0.18–0.34, *P* = 0.023, I^2^ = 61.8%), 41% with PHQ-9 (95% CI: 0.20–0.63, *P* < 0.001, I^2^ = 90.7%), and 37% with other tools such as BSI, BDI, and CES-D (95% CI: 0.21–0.53, *P* = 0.031, I^2^ = 71.3%). Furthermore, the Egger's test indicates that publication bias is insignificant among the studies included. For secondary outcome, pooled results indicated that the prevalence of anxiety among cSLE patients was 29.0% (95% CI: 0.19–0.42, *P* < 0.001), exhibiting relatively high heterogeneity (I^2^ = 61.8%, *τ*^2^ = [0.13] on the logit scale)

**Conclusions:**

Approximately one-third of patients with cSLE screened positive for depressive symptoms, underscoring the critical need for routine mental health screening in clinical care. These prevalence estimates should be interpreted with caution due to methodological and clinical heterogeneity across studies and do not reflect the prevalence of clinically diagnosed major depressive disorder.

## Introduction

Systemic lupus erythematosus (SLE) is a chronic, inflammatory, multi-system autoimmune disease characterized by its involvement of multiple organs, including the kidneys, lungs, and nervous system. Numerous studies have demonstrated that SLE is also associated with psychological manifestations, including depression and anxiety (1–3), collectively termed neuropsychiatric systemic lupus erythematosus (NPSLE). Moreover, several investigations have reported considerable variability in the prevalence of NPSLE across different populations and geographic regions (4–6). In the pediatric population, cSLE frequently presents with more severe psychological symptoms, particularly depressive moods that can lead to significant psychiatric sequelae, disability, and increased mortality rates (7–9). Consequently, cSLE complicated by depression represents a critical issue in clinical practice that necessitates greater attention to identify more effective treatment strategies.

For children diagnosed with SLE who exhibit depressive symptoms, there exists a limited number of reported literature. Furthermore, the findings regarding prevalence and disease course are inconsistent. In light of the prevailing gaps in our understanding, this meta-analysis aimed to estimate the pooled prevalence of screening-positive depressive symptoms among patients with cSLE and to explore variation in prevalence according to the screening instruments used. Meanwhile, anxiety were included as secondary objectives.

## Methods

This systematic review and meta-analysis was conducted in accordance with the Meta-analyses of Observational Studies in Epidemiology (MOOSE) guidelines (10) (see Supplementary Table S1) and the Preferred Reporting Items for Systematic Reviews and Meta-Analyses (PRISMA) statement and checklist (11) (see Supplementary Table S2).

### Searching strategy

A total of three prominent medical databases, from their inceptions to 1 Nov, 2024 were employed for the literature search: PubMed, Embase, and the Cochrane Library. Medical Subject Headings (MeSH) in conjunction with pertinent free-text keywords (synonyms) were utilized in various combinations to enhance the effectiveness of the search process (Supplementary Materials). The key-words included “systemic lupus erythematosus,” “children,” and “depression.” The literature search imposed no restrictions regarding language, region, ethnicity, or country.

### Definition of cSLE and depression/anxiety symptoms

For this review, cSLE was defined as SLE with disease onset prior to age 18. As this review specifically addressed depressive symptoms in pediatric and adolescent populations, eligible participants were required to be under 18 years of age at the time of depression assessment.

We define cases as participants screening positive for depressive/anxiety symptoms according to the instrument and cut-off score used in each original study, rather than as participants with a clinically confirmed diagnosis of depressive.anxiety disorder.

### Literature selection

This systematic review and meta-analysis have **inclusion and exclusion criteria** for the selection of literature, delineated as follows:

**Inclusion Criteria:**

1) Studies reporting data on children and adolescents younger than 18 years at the time of depression assessment who had SLE onset before 18 years of age;
2) Studies employing validated screening tools for the assessment of depression symptoms;
3) Studies designed as cross-sectional, retrospective, or prospective cohorts;
4) Inclusion of patients who meet the revised American College of Rheumatology or Systemic Lupus Collaborating Clinics Classification Criteria for SLE;
5) Patients diagnosed with NPSLE with anxiety were also included as secondary analyses.
6) Studies were included without publication language restrictions.

**Exclusion Criteria:**

1) Studies lacking a validated screening tool for the diagnosis of depression;
2) Studies that do not specify diagnostic criteria for SLE;
3) Non-human studies, including animal research, laboratory investigations, and *in vitro* experiments;
4) Studies with insufficient data, particularly those presenting information solely in an abstract form.

### Literature screening process

Stringent principles and methodologies of quality control were meticulously applied throughout the literature screening process. The comprehensive literature screening procedure was thoroughly documented. Two independent reviewers (XJX and MP) conducted this search process. In cases where discrepancies arose, they engaged in collaborative discussions to reach a resolution.

### Data extraction

The study employed a pre-established Excel spreadsheets for data extraction. The extracted information encompassed the first author's name, year of publication, study period, age, gender, sample size, withdrawals, dropouts, follow-up duration, and outcome measures. The primary outcome measure was defined as the prevalence of depression in patients with cSLE, while the secondary outcome measures included mean depression scores, the course of depression and associated risk factors.

### Methodological quality evaluation

The methodological quality of the publications included in this review was assessed using the Newcastle-Ottawa Scale (12), which allocates a maximum quality score of 9 points. Two authors independently evaluated the methodological quality and calculated the average as the final score.

### Statistical methods

Odds ratios (OR) accompanied by 95% confidence intervals (CI) were employed as the effect sizes for the meta-analysis. Study-specific proportions were logit-transformed and pooled using a random-effects model. Between-study variance (*τ*^2^) was estimated using restricted maximum likelihood (REML), and the Hartung–Knapp-Sidik-Jonkman adjustment was used to calculate 95% confidence intervals for the pooled logit proportion. Pooled estimates and confidence limits were back-transformed to the proportion scale. The DerSimonian-Laird estimator with conventional normal-based inference was used in sensitivity analyses. Specifically, an I^2^ value ranging from 0 to 25% signifies negligible heterogeneity, while values between 26% and 50% indicate low heterogeneity; those spanning from 51% to 75% denote moderate heterogeneity, and values exceeding 75% are indicative of high heterogeneity. A sensitivity analysis was executed to uncover potential sources of variability in study outcomes. Furthermore, funnel plots alongside Egger's test were employed to scrutinize publication bias across the studies examined. Statistical analyses were performed using STATA software, with a two-tailed *P*-value of less than 0.05 denoting statistical significance.

## Results

### Study selection process

The initial search resulted in a total of 661 records obtained from three medical databases: PubMed (306 records), Embase (299 records), and the Cochrane Library (56 records). Following the removal of duplicates and the exclusion of publications reporting irrelevant topics, 72 articles were selected for full-text screening. Ultimately, 12 eligible articles (7, 13–23) were incorporated into the final pooled analysis. A detailed flow diagram is provided in Figure 1.

**Figure 1:**
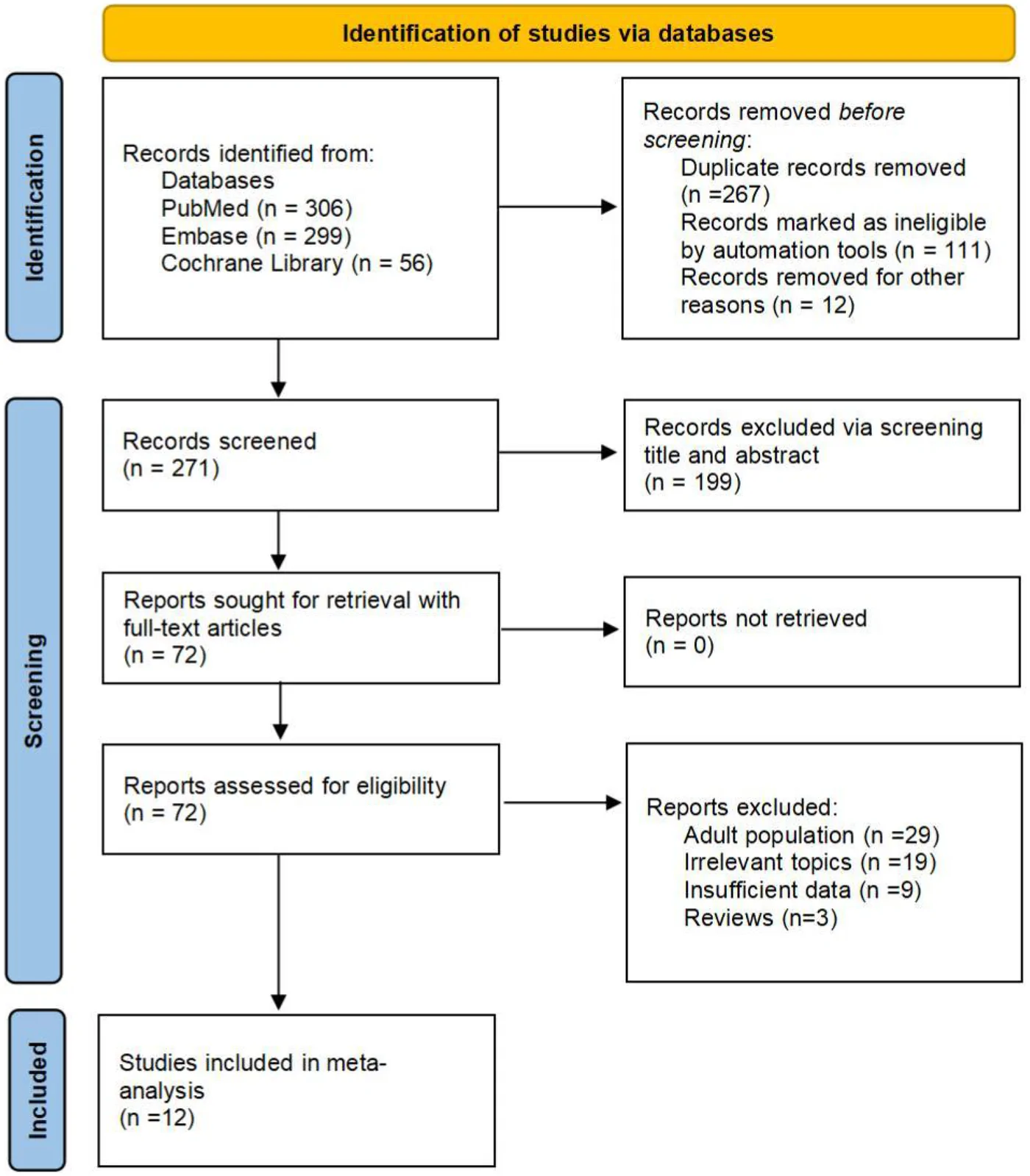
Flow chart of study selection in accordance with the PRISMA guidelines.

### Characteristics of included studies

As presented in detail in Table 1, the pooled analysis included a total of 12 articles encompassing 652 cSLE patients. All articles are published in English, with publication years spanning from 2010 to 2024. The sample sizes of the studies included in this analysis vary, ranging from 24 to 106. All diagnostic criteria for SLE as delineated in included literatures conform to the guidelines established by the American College of Rheumatology (ACR) 1997. The criteria for diagnosing depression are not uniform, including the Childhood Depression Inventory (CDI): 6 articles; Patient Health Questionnaire-9 (PHQ9): 3 articles; and others (Brief Symptom Inventory/Beck Depression Inventory/Birleson Scale/Center for Epidemiologic Studies Depression Scale for Children): 3 articles.

**Table 1:** Comprehensive characteristics of the included articles (*N* = 12).

First Author	Country	Language	Publication Year	Study Period	Study Design	Sample size	Age (years)	Gender (Male %)	Depression validated tool	Criteria for SLE
S Ahola Kohut	Canada	English	2013	2010–2012	Cross-sectional	38	14.3 ± 2.4	6 (16%)	CDI	ACR
Catherine Donnelly	US	English	2018	NR	Cross-sectional	50	16.2 ± 2.5	8 (16%)	CDI	ACR
Eyal Muscal	US	English	2010	2001–2008	Retrospective + Prospective Cohort	39	RC: 15.1 (12.2–16.9) PC: 15.8 (14.8–16.5)	RC: 3 (12.5%) PC: 2 (13.3%)	CDI	ACR
Jordan Jones	US	English	2016	NR	Cross-sectional	60	16.1 ± 2.5	11 (20%)	CDI	ACR
Yasemin Düzçeker	Turkey	English	2013	2011–2012	Cross-sectional	25	NR	NR	BSI	ACR
Tricia S. Williams	Canada	English	2011	2006–2009	Case-control study	41	16.4 ± 2.5 (10.3- 21.8)	11 (27%)	BDI	ACR
Li Ji	China	English	2012	2010–2011	Case-control study	84	15.3 ± 1.4	0 (0%)	CDI	ACR
Alaina M. Davis	US	English	2018	2016	Cross-sectional	51	16.2 ± 3.3 (7–22)	4 (7.8%)	PHQ-9	ACR
H Aldar	Brazil	English	2012	2010	Retrospective Cohort	50	16.82 ± 3.46	3 (5.7%)	CDI	ACR
Kate M. Neufeld	Canada	English	2024	2017–2019	Retrospective Cohort	51	15.0 ± 1.9	6 (12%)	CES-DC	ACR
Tamar B. Rubinstein	US&Canada	English	2023	2017–2020	Multicenter, retrospective cohort	106	Median:17 IQR:15–19	12 (11%)	PHQ-9	ACR
Evan Mulvihill	US	English	2021	2017	Retrospective Cohort	62	13.32 + 3.04	9 (14.5%)	PHQ-9	ACR

US, United States; NR, Not Report; RC, retrospective cohort; PC, Prospective cohort; CDI, Childhood Depression Inventory; BSI, Brief Symptom Inventory; BDI, Beck Depression Inventory; CES-DC, Center for Epidemiologic Studies Depression Scale for Children; PHQ-9, Patient Health Questionnaire-9; ACR, 1997 American College of Rheumatology revised criteria for the classification of systemic lupus erythematosus.

### Prevelance of depression symptoms in cSLE patients

Twelve studies reported the prevalence of depressive symptoms among patients with cSLE. In the primary analysis, logit-transformed proportions were pooled using a random-effects model with restricted maximum likelihood (REML) estimation and the Hartung-Knapp-Sidik-Jonkman adjustment. The pooled prevalence was [32.6%] (95% CI: [24.5%–41.8%]; Figure 2). Between-study heterogeneity was moderate (I^2^ = [73.66%]; *τ*^2^ = [0.26] on the logit scale). In a sensitivity analysis using the DerSimonian-Laird estimator with conventional normal-based inference, the pooled prevalence was approximately 33% (95% CI: 26%–40%) Figure 2.

**Figure 2:**
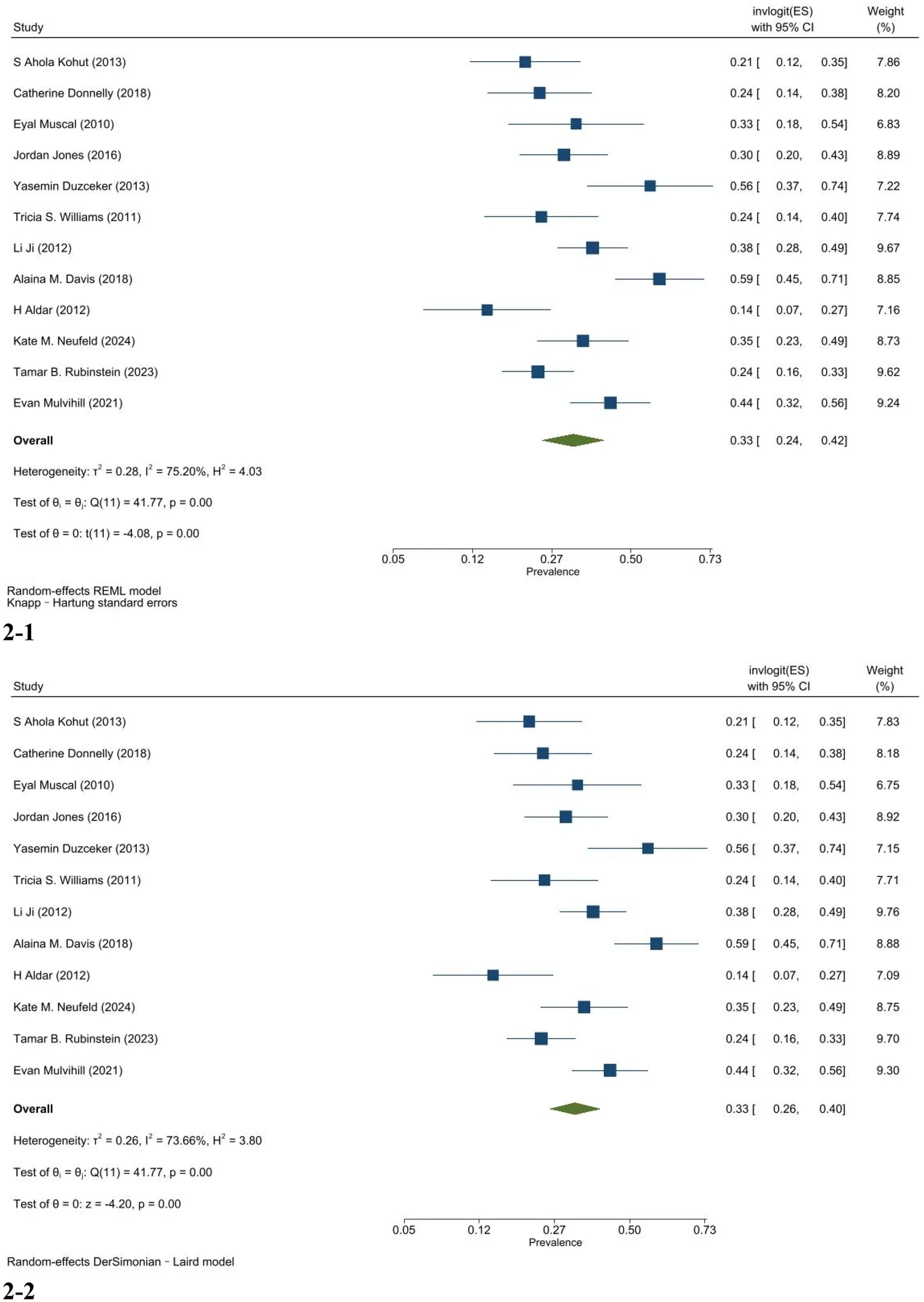
Pooled results for prevelance of depression in cSLE.

### Subgroup analysis of depression prevalence based on different screening tools

Subgroup analysis of depression prevalence was performed based on various screening tools. A total of 6 studies reported depression prevalence using CDI, while 3 studies utilized the PHQ-9. Additionally, 3 studies employed other assessment instruments, including the Brief Symptom Inventory (BSI), Beck Depression Inventory (BDI), and Center for Epidemiologic Studies Depression Scale (CES-D).

As demonstrated in Figure 3, the subgroup analysis indicated that the prevalence of depression varied according to the different screening tools used: it was found to be 26% with CDI (95% CI: 0.18–0.34, *P* = 0.023, I^2^ = 61.8%), 41% with PHQ-9 (95% CI: 0.20–0.63, *P* < 0.001, I^2^ = 90.7%), and 37% with other tools such as BSI, BDI, and CES-D (95% CI: 0.21–0.53, *P* = 0.031, I^2^ = 71.3%).

**Figure 3:**
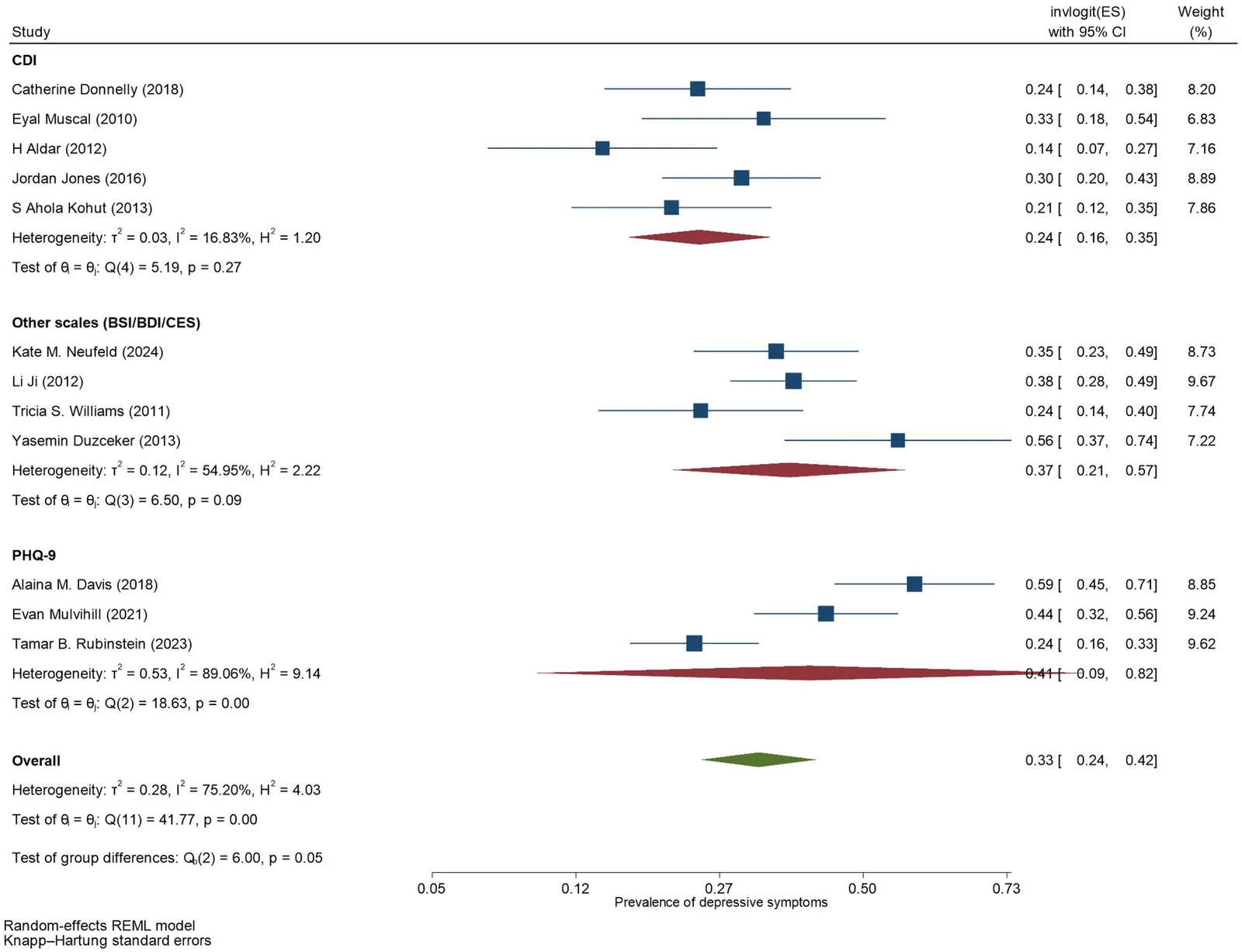
Subgroup meta-analysis of the prevalence of depressive symptoms in patients with cSLE by screening instrument.

### Secondary outcome on anxiety

There were 5 studies reported anxiety symptoms, The pooled results indicated that the prevalence of anxiety among cSLE patients was 29.0% (95% CI: 0.19–0.42, *P* < 0.001), exhibiting relatively high heterogeneity (I^2^ = 61.8%, *τ*^2^ = [0.13] on the logit scale), as illustrated in Figure 4.

**Figure 4:**
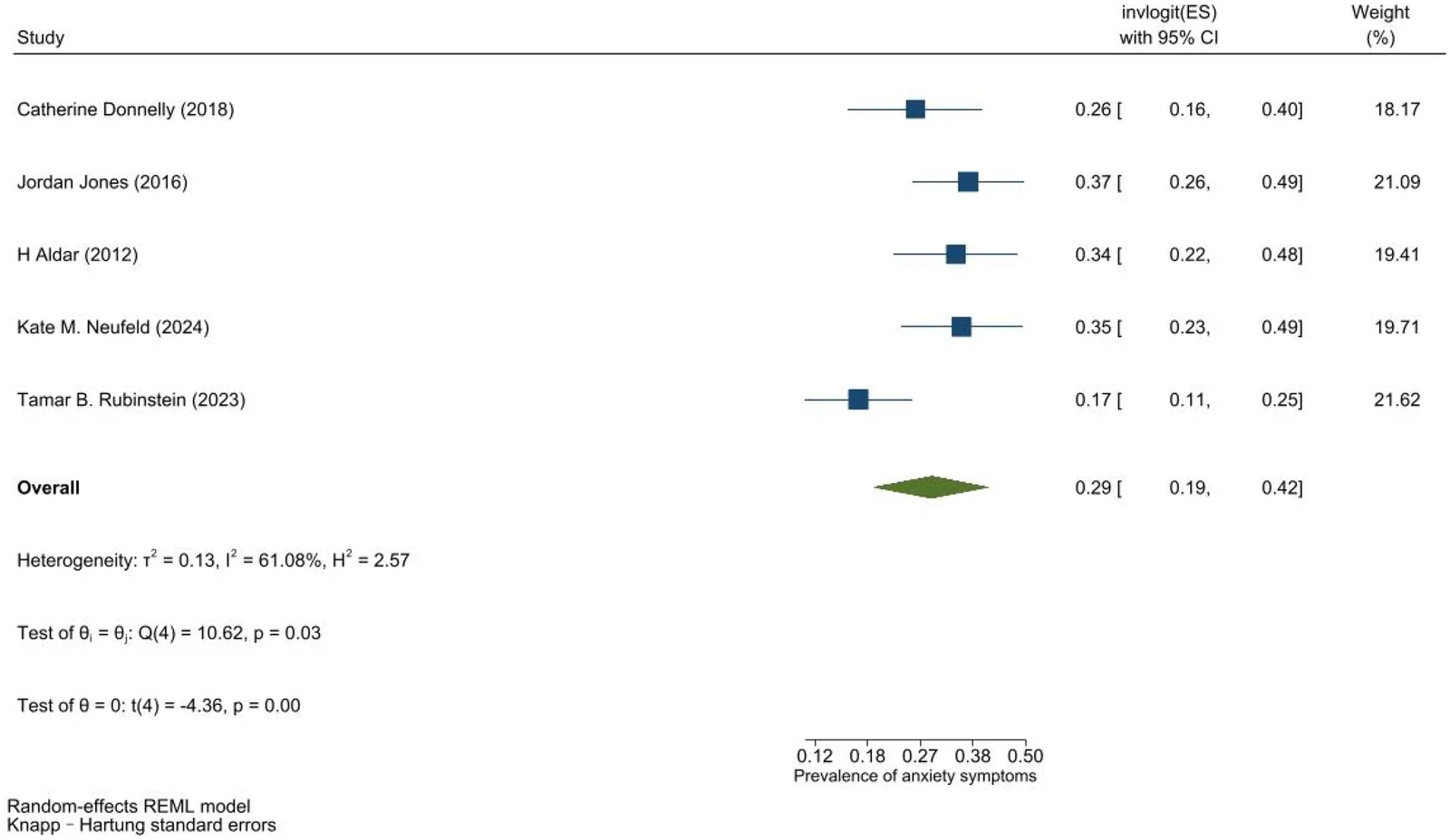
Pooled results for prevelance of anxiety in cSLE.

### Exploration of potential sources of heterogeneity with sensitivity analysis

As illustrated in Figure 5, the subsequent sensitivity analysis did not identify any potential sources of heterogeneity across the studies. The variations among the included studies remained stable, situated between the lower and upper confidence interval limits.

**Figure 5:**
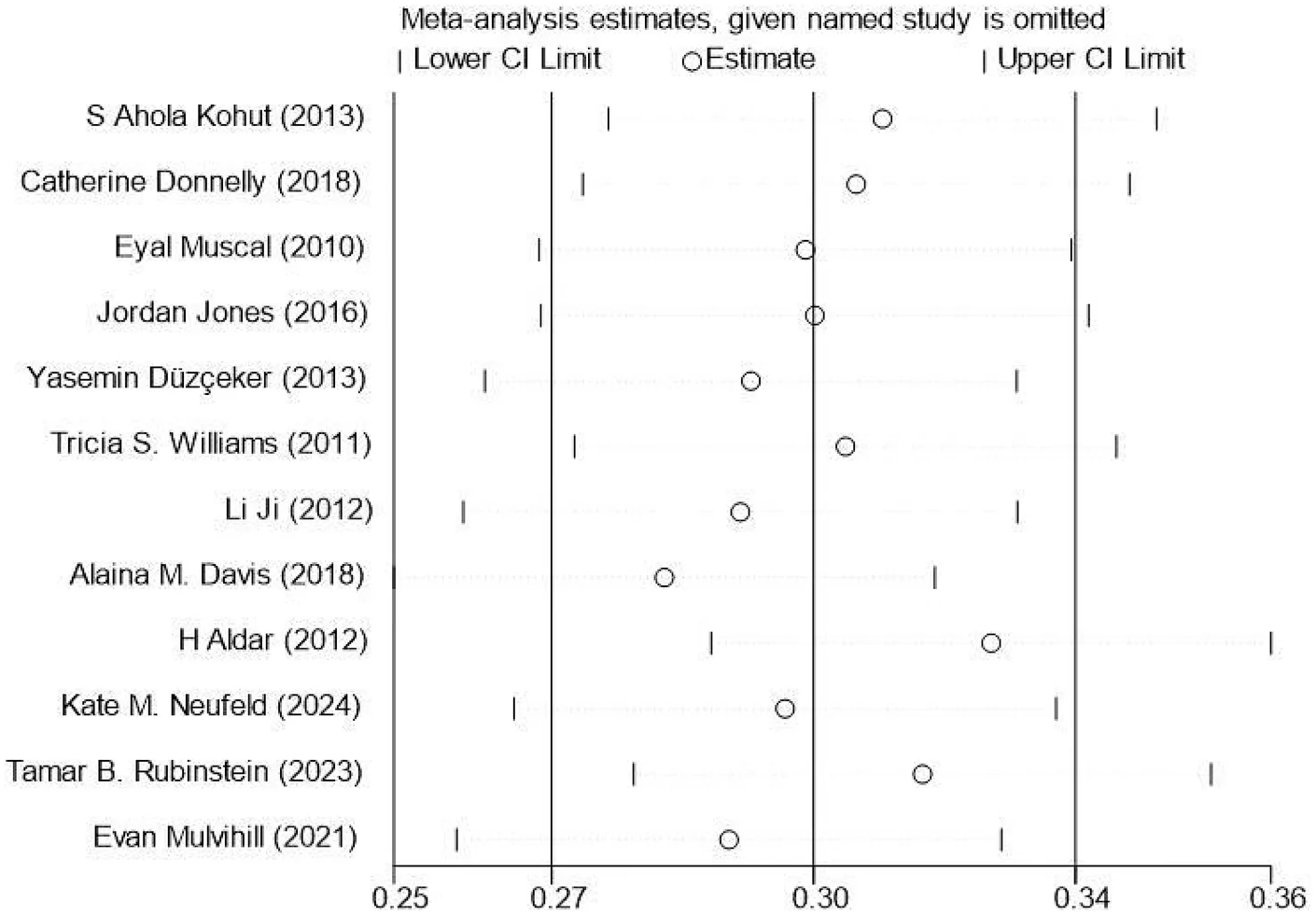
Sensitivity analysis to investigate the potential sources of heterogeneity.

### Methodological quality and publication bias assessment

As illustrated in Table 2, which presents the assessment of methodological quality, each publication included demonstrated commendable methodological rigor. The Newcastle-Ottawa scores were all no less than 6, indicating a relatively high standard of methodological quality across the studies.

**Table 2:** Assessment of methodological qualities utilizing the Newcastle-Ottawa scale.

First Author		Selection(0–4)		Comparability(0–2)	Outcome(0–3)		Outcome(0–3)			Total
	REC	SNEC	AE	DO	SC	AF	AO	FU	AFU	
S Ahola Kohut	1	1	1	0	1	0	1	1	1	7
Catherine Donnelly	1	1	1	1	1	0	1	1	1	8
Eyal Muscal	1	1	1	0	1	1	1	1	1	8
Jordan Jones	1	1	1	1	0	0	1	1	1	7
Yasemin Düzçeker	1	0	1	1	0	1	1	1	1	7
Tricia S. Williams	1	1	1	1	0	0	1	1	1	7
Li Ji	1	1	1	1	0	0	1	1	1	7
Alaina M. Davis	1	1	1	1	0	1	1	1	1	8
H Aldar	1	1	1	1	0	0	1	1	1	7
Kate M. Neufeld	1	1	1	1	0	1	1	1	1	8
Tamar B. Rubinstein	1	1	1	1	0	0	1	1	1	7
Evan Mulvihill	1	1	0	1	1	1	1	1	1	8

“1” indicates that the cohort study has met the specified criterion, while “0” denotes a failure to do so.

REC, Representativeness of the exposed cohort; SNEC, Selection of the non-exposed cohort; AE, Ascertainment of exposure; DO, Demonstration that the outcome of interest was not present at the start of the study; SC, Study controls for age and sex; AF, Study controls for any additional factors (such as donor source, immunotherapy, etc.); AO, Assessment of outcome; FU, Follow-up duration sufficient (36 months) for outcomes to manifest; AFU, Adequacy of follow-up among cohorts (≥90%).

As demonstrated in Figures 6A,B, the Egger's test indicates that publication bias is insignificant among the studies included, with *P*-value > 0.05.

**Figure 6:**
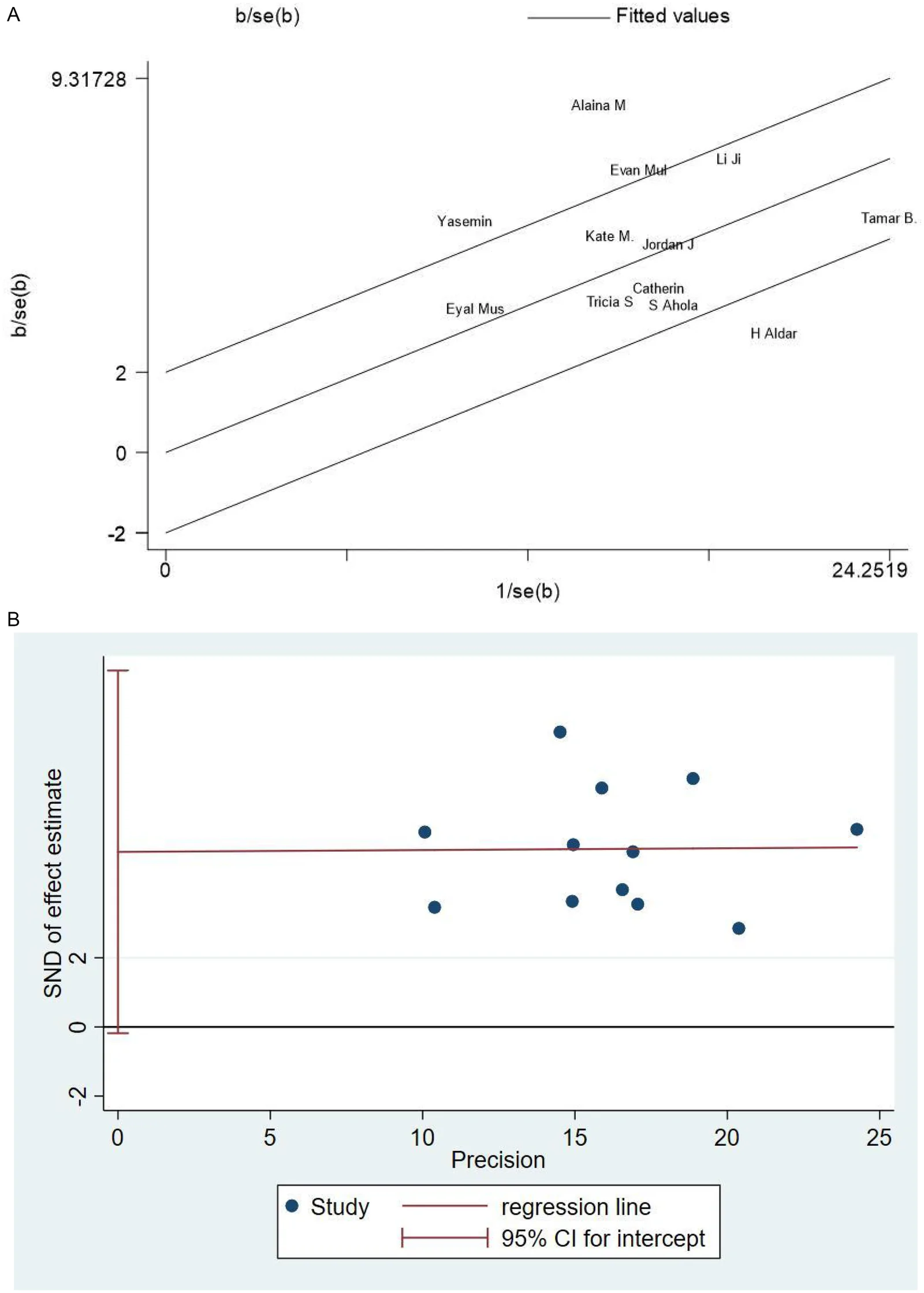
**(A)** assessments of publication bias with Egger's test **(B)**.

## Discussions

This systematic review and meta-analysis estimated the pooled prevalence of screening-positive depressive symptoms among patients with cSLE. The pooled prevalence of screening-positive depressive symptoms was 32.6%. The pooled prevalence of screening-positive depressive symptoms was 26% in studies using the CDI, 41% in those using the PHQ-9, and 37% in those using other instruments. These numerical differences should be interpreted cautiously because of between-study heterogeneity and differences in study populations and screening thresholds. Our investigation also delved into potential sources of heterogeneity; however, no distinct heterogeneous literature emerged from our analysis. Additionally, all NOS scores pertaining to our methodological evaluation exceeded a score of 6, indicating high-quality literatures. The Egger's test further confirmed that there was no significant publication bias present in our findings.

Beyond differences in screening instruments, several clinical and contextual factors may contribute to variation in the reported prevalence of depressive symptoms among patients with cSLE. Regional differences in healthcare access, mental health services, cultural attitudes toward reporting psychological symptoms, and study recruitment settings may affect both the identification and reporting of depressive symptoms. Disease-related factors, including disease activity, cumulative organ damage, and neuropsychiatric manifestations, may also be relevant. In addition, comorbid conditions and symptoms such as chronic pain, fatigue, and sleep disturbance could influence depressive symptom scores. Treatment-related factors, particularly glucocorticoid exposure and treatment burden, warrant consideration. However, these factors were not reported consistently across the included studies, and the available aggregate data did not permit reliable subgroup analyses or meta-regression. Therefore, their contributions to the observed heterogeneity remain uncertain and should not be inferred from the present findings. Future multicenter studies should use consistent depression assessment criteria and report these clinical and contextual variables to allow a more informative examination of potential determinants of depressive symptoms in cSLE.

A previous meta-analysis conducted by Lijuan Zhang et al. (24). indicated that the crude prevalence of SLE among adults was 24%. Consequently, our pooled results demonstrate that the prevalence among patients with cSLE is slightly higher than that observed in adult patients. Nevertheless, owing to the disparities in measurement scales, the outcomes of our comparative analysis with adults must be approached and interpreted with a degree of caution. Therefore, this meta-analysis provides pooled estimate of the prevalence of screening-positive depressive symptoms among patients with cSLE, which should be interpreted in light of the methodological and clinical heterogeneity across studies.

Variation in prevalence estimates across screening instruments highlights the importance of clearly reporting the instruments and cut-off scores used to define screening-positive depressive symptoms. Future studies using consistent, age-appropriate assessment methods and clearly defined thresholds, would improve comparability across studies, as well as strengthen estimates of depressive symptom prevalence in cSLE population.

### Functional outcomes, quality of life, and treatment adherence

Depressive symptoms in cSLE may be associated with poorer daily functioning and health-related quality of life (15). Evidence from broader SLE populations also suggests an association with medication adherence, although its applicability to cSLE requires further investigation (25). These outcomes were inconsistently reported in the included studies, preventing quantitative assessment of their associations with depressive symptoms or causal inference. Longitudinal studies using validated measures are needed.

### Clinical implications

Our findings support considering age-appropriate depression screening in routine cSLE care, consistent with guidance for adolescent depression assessment (26). Positive screening results require clinical assessment and, when indicated, mental health referral (26). Tool selection should consider patient age, local validation, feasibility, and follow-up resources. As this review did not compare diagnostic accuracy, no instrument can be recommended as superior; population-specific validation is needed (27, 28).

### Strengths and limitations of this study

In addition, the present systematic review and meta-analysis exhibits both strengths and limitations. In terms of strengths, this investigation represents the inaugural pooled analysis concerning the prevalence of depression in cSLE patients and its disease course, to the best of our knowledge. Furthermore, the studies included in this review demonstrated relatively high methodological rigor, as evidenced by Newcastle-Ottawa Scale (NOS) scores that were no less than 6. Additionally, we conducted a subgroup analysis which revealed significant discrepancies in the assessment of depression prevalence contingent upon varying screening tools for depression. This finding holds substantial implications for clinical practice and nursing, offering valuable insights into tailored approaches for patient care.

This review has several limitations. First, the small number of included studies and their limited sample sizes reduce the precision and generalizability of the pooled estimate. Second, heterogeneity across studies was substantial, likely attributable to differences in screening instruments, cut-off thresholds, and characteristics of the study populations. Third, the pooled prevalence reflects screening-positive depressive symptoms, not clinically diagnosed depressive disorders, thereby limiting its clinical interpretability. Fourth, inconsistent reporting of participants' clinical characteristics and prior treatment exposure hindered a robust exploration of potential sources of heterogeneity. Fifth, as all evidence is observational, causal inferences cannot be drawn. Finally, although Egger's test for publication bias was nonsignificant, such bias cannot be definitively ruled out due to the limited number of studies.

## Conclusions

Approximately one-third of cSLE patients screened positive for depressive symptoms in this meta-analysis. Although prevalence estimates varied across individual studies and screening instruments, these findings underscore a substantial burden of depressive symptoms and reinforce the need for routine mental health assessment in clinical care for children and adolescents with cSLE. Importantly, a positive screening result does not constitute a clinical diagnosis of depressive disorder and must be followed by comprehensive clinical evaluation.

## Data Availability

The original contributions presented in the study are included in the article/Supplementary Material, further inquiries can be directed to the corresponding author/s.
