# Supplementary material for: Prevalence of depression in childhood-onset systemic lupus erythematosus: a systematic review and meta-analysis
Source: Front Pediatr. 2026 Oct 29;14:1966337. doi: 10.3389/fped.2026.1966337 (PMC13755206; doi:10.3389/fped.2026.1966337)
Supplement: Supplementary file 1 [file Supplementaryfile1.docx]

PubMed:

| Search number | Search terms |
| --- | --- |
| #1 | "Lupus Erythematosus, Systemic"[Mesh] OR "systemic lupus erythematosus"[tiab] OR lupus[tiab] OR SLE[tiab] OR cSLE[tiab] OR jSLE[tiab] |
| #2 | "Infant"[Mesh] OR "Child"[Mesh] OR "Adolescent"[Mesh] OR infant*[tiab] OR child*[tiab] OR adolescen*[tiab] OR pediatric*[tiab] OR paediatric*[tiab] OR juvenile*[tiab] OR teen*[tiab] OR youth*[tiab] OR preschool*[tiab] OR "school age"[tiab] OR "school aged"[tiab] OR "childhood onset"[tiab] OR "pediatric onset"[tiab] OR "paediatric onset"[tiab] OR "juvenile onset"[tiab] |
| #3 | "Depression"[Mesh] OR "Depressive Disorder"[Mesh] OR depress*[tiab] |
| #4 | #1 AND #2 AND #3 |

Embase:

| Search number | Search terms |
| --- | --- |
| #1 | 'systemic lupus erythematosus'/exp OR 'systemic lupus erythematosus':ti,ab,kw OR lupus:ti,ab,kw OR sle:ti,ab,kw OR csle:ti,ab,kw OR jsle:ti,ab,kw |
| #2 | 'infant'/exp OR 'child'/exp OR 'adolescent'/exp OR infant*:ti,ab,kw OR child*:ti,ab,kw OR adolescen*:ti,ab,kw OR pediatric*:ti,ab,kw OR paediatric*:ti,ab,kw OR juvenile*:ti,ab,kw OR teen*:ti,ab,kw OR youth*:ti,ab,kw OR preschool*:ti,ab,kw OR 'school age':ti,ab,kw OR 'school aged':ti,ab,kw OR 'childhood onset':ti,ab,kw OR 'pediatric onset':ti,ab,kw OR 'paediatric onset':ti,ab,kw OR 'juvenile onset':ti,ab,kw |
| #3 | 'depression'/exp OR depress*:ti,ab,kw |
| #4 | #1 AND #2 AND #3 |

Cochrane Library:

| Search number | Search terms |
| --- | --- |
| #1 | MeSH descriptor: [Lupus Erythematosus, Systemic] explode all trees |
| #2 | ("systemic lupus erythematosus" OR lupus OR SLE OR cSLE OR jSLE):ti,ab,kw |
| #3 | #1 OR #2 |
| #4 | MeSH descriptor: [Infant] explode all trees |
| #5 | MeSH descriptor: [Child] explode all trees |
| #6 | MeSH descriptor: [Adolescent] explode all trees |
| #7 | (infant* OR child* OR adolescen* OR pediatric* OR paediatric* OR juvenile* OR teen* OR youth* OR preschool* OR "school age" OR "school aged" OR "childhood onset" OR "pediatric onset" OR "paediatric onset" OR "juvenile onset"):ti,ab,kw |
| #8 | #4 OR #5 OR #6 OR #7 |
| #9 | MeSH descriptor: [Depression] explode all trees |
| #10 | MeSH descriptor: [Depressive Disorder] explode all trees |
| #11 | depress*:ti,ab,kw |
| #12 | #9 OR #10 OR #11 |
| #13 | #3 AND #8 AND #12 |
